# Formononetin Inhibits Hepatic I/R-Induced Injury through Regulating PHB2/PINK1/Parkin Pathway

**DOI:** 10.1155/2022/6481192

**Published:** 2022-12-02

**Authors:** Zhongying Ma, Di Zhang, Jin Sun, Qi Zhang, Yi Qiao, Yixin Zhu, Jing Niu, Qian Ren, Lun Zhou, Aidong Wen, Jingwen Wang

**Affiliations:** Department of Pharmacy, Xijing Hospital, Fourth Military Medical University, Xi'an, 710032 Shaanxi, China

## Abstract

Formononetin (FN), an isoflavone compound mainly isolated from soy and red clover, had showed its anti-inflammation, antioxidative effects in some degenerative diseases and cholestasis. However, the role of FN in protecting ischemia/reperfusion- (I/R-) induced liver injury and the possible mechanism were unclear. In this study, effects of FN on liver injury were investigated in a rat hepatic I/R model; further, mitophagy-related proteins were measured by immunoblotting or immunofluorescence. The possible roles of PHB2 and PINK1 in regulating mitophagy by FN were verified using adeno-associated virus knockdown. The results showed that FN had protective effects against hepatic I/R injury through regulating PINK1/Parkin-regulated mitophagy. Further, we found that FN inhibited PARL expression and prevented PGAM5 cropped by increasing the expression of PHB2. The knockdown of PINK1 or PHB2 both abolished the protective effects of FN. Taken together, our findings indicated that the isoflavone compound FN promoted PHB2/PINK1/Parkin-mediated mitophagy pathway to protect liver from I/R-induced injury. These results provided novel insights into the potential prevention strategies of FN and its underlying mechanisms.

## 1. Introduction

During some hepatic surgeries like liver resection, transplantation, and some trauma repairs, the blood supplement to the liver will always be temporarily cut off and restored after operations, thus leading to ischemia/reperfusion (I/R) [1]. For example, liver transplantation is a standard surgery for the treatment of end-stage liver disease; however, temporary vascular occlusion will initiate liver injuries and aggravated when reperfusion [2]. Even the surgical techniques were improved greatly, and hepatic I/R accounts for about 10% acute graft dysfunction in liver transplantation and leads to the failure of transplantation in the first year, which need to repeat transplantation and cause further shortages of liver donors [3]. In the context of the huge amounts of liver disease all over the world which need to liver resection and/or transplantation, hepatic I/R remains an urgent clinical problem to be solved [4]. Despite these urgently needs, few available strategies have been proven effective in the treatment of hepatic I/R. Thus, to prevent and treat hepatic I/R injury, parts of clinical medicines or native compounds should be screened and developed to excavate the potential bioactive components.

Liver is the largest organ in the human body and plays important role in regulating the body's metabolic. These processes consume huge amounts of energy which produced by mitochondria from oxygen and glycolipids [5]. When liver is subjected to ischemia and hypoxia, the mitochondria function is destroyed, and the hepatic functions are damaged [6]. During I/R, the structure of mitochondria is damaged, accompanied with the decrease of adenosine triphosphate (ATP) production, mitochondria-related apoptosis, and mitochondria fission [7]. Dysfunction mitochondria produce redundant reactive oxygen species (ROS) which induce damage to DNA, proteins, and lipid and also release damage-associated molecular pattern molecules which aggravate the inflammatory [8]. Thus, it is necessary to clear the dysfunction mitochondria to maintain cellular homeostasis. Mitophagy, a selective mitochondria autophagy, plays a very important role in removing damaged mitochondria [9]. Many studies had showed that mitophagy was impaired during I/R, leading to the inadequate capability to remove damaged mitochondria in the reperfused hepatocytes [10]. Above all, resuming the capability of mitophagy has protective effect against I/R-induced hepatic injuries.

The PTEN-induced putative kinase protein 1- (PINK1-) Parkin-mediated mitophagy has been best described in the previous studies [11, 12]. In the basal conditions, PINK1 imports across the outer mitochondrial membrane (OMM) and degraded by PARL [13]. Upon stimulation, PINK1 cannot be processed by PARL and recruits Parkin resulting in the polyubiquitination of some proteins on OMM which have important effects on autophagy [14]. Several studies have found the activation of PINK1/Parkin-dependent mitophagy plays a protective role against I/R-induced liver injury [15]. Comprehensively, considering the mitophagy-mediated dysfunction mitochondrial clearance inhibits hepatic I/R injury, activating the PINK1/Parkin pathway is a potential strategy in the treatment of ischemia hepatic injury.

Isoflavones are a group of phytoestrogens with several health benefits, including antioxidant, apoptotic, anti-inflammatory, and some other biological functions, and they have got the attention of researchers in the areas of nutrition, medicine, and other health products [16]. Formononetin (FN), a kind of isoflavone, is mainly isolated from soy and red clover, and it has performed its biological effects on some degenerative diseases, like hypertension, diabetes, osteoporosis, and degenerative brain diseases [17]. Recently, studies showed that FN had protective effects against drug-induced hepatotoxicity, liver inflammatory lesions, cholestasis, and hepatic steatosis [17–19], indicating its benefit effects in treating liver diseases. In a concanavalin A-induced hepatitis model, FN showed inhibition effects on inflammation through inhibiting nuclear factor kappa B (NF-*κ*B) and NOD-like receptor protein 3 (NLRP3) inflammasome pathway [18]. In cerebral I/R animal models, FN showed its inhibition effects on apoptosis through activation of PI3K/Akt signaling pathway and downregulation of Bax/Bcl-2 ration [20, 21]. Also, FN inhibited myocardial cell I/R-induced cellular apoptosis in aged cells by facilitating autophagy [21, 22]. However, the effects of FN on hepatic I/R were largely unknown to us. In this study, the protective effects of FN were evaluated in a hepatic I/R rat model, and further, the possible mechanisms were investigated.

## 2. Materials and Methods

### 2.1. Reagents and Compounds

Formononetin (FN) with a purity > 98% identified by HPLC was obtained from Herbest Biotechnology Co., LTD. ALT, AST, TNF-*α*, and IL-1*β* measurement kits were obtained from Beyotime Biotechnology. TUNEL Apoptosis Detection Kit was obtained from YEASEN Biotechnology. MDA, ROS, GSH, CAT, and GSH-Px measurement kits were provided by Beyotime Biotechnology. LC3 II, P62, Beclin1, COX2, and COX4 primary antibodies were obtained from Abcam. TOM20, Parkin, PINK1, PHB2, PGAM5, and PARL primary antibodies were produced by Cell Signaling Technology. 3-Methyladenine was obtained from Sigma-Aldrich. Other reagents used in the study were obtained from local commercial suppliers.

### 2.2. Animals and Groups

Male rats (200-220 g) were obtained from the Experimental Animal Center of Fourth Military Medical University (Xi'an, China). All rats were raised with free water and food in a temperature controlled and specific pathogen-free room. All the operations based on the ethics committee on animal experiments were reviewed and approved by the Animal Care and Use Committee of Fourth Military Medical University. To induce I/R injury model, the rats were anesthetized by pentobarbital (40 mg/kg) intraperitoneally. The hepatic artery and portal vein were inserted by noninvasive arterial clamps to block blood supply according to the method reported [23]. After ischemia for 1 h, the clamps were removed and reperfused. After reperfusion for 6 h, the drugs or buffer were given to rats. During the treatments, rats were free to food and water.

To study the effects of FN on the I/R-induced liver injury, 50 rats were randomly divided into 5 groups: control group (*n* = 10, rats were laparotomized and abdomen closured without I/R surgery), model group (*n* = 10, rats were subjected to I/R surgery and administered intragastrically with saline solution after I/R), FN-L group (*n* = 10, rats were subjected to I/R surgery and administered intragastrically with 30 mg/kg FN), FN-M group (*n* = 10, rats were subjected to I/R surgery and administered intragastrically with 60 mg/kg FN), and FN-H group (*n* = 10, rats were subjected to I/R surgery and administered intragastrically with 90 mg/kg FN). The solvent buffer and FN were given after 6 h for reperfusion, twice each day for one week. After those, liver tissues and blood samples were collected and stored at -80°C until use. FN had been reported for its protective effects against acetaminophen- (APAP-) induced liver injury at 100 mg/kg [24], and considering this and our preliminary experiment results, 30, 60, and 90 mg/kg were determined to be used in further studies.

### 2.3. Plasma Biochemical Measurements

For biochemical measurements, serum from different groups was collected after blood samples centrifuged at 3000 g for 15 min. Alanine aminotransferase (ALT) catalyzes the amino conversion reaction between alanine and *α*-ketoglutaric acid and results in a colorimetric product which can be measured by a microplate reader. Aspartate transaminase (AST) catalyzes the amino conversion between aspartic acid and *α*-ketoglutaric acid and results in a colorimetric product [25]. TNF-*α* and IL-1*β* in the serum were measured by the specific enzyme-linked immune-absorbent assay kits. All the operations were according to the manuscript of the kits.

### 2.4. Histochemical Analysis and Masson Staining

After different treatments, the liver tissues were collected and stored in the 10% formalin. The liver tissue samples were embedded with paraffin, cut to 5 *μ*M sections, and stained with hematoxylin-eosin. The slides were assessed as the criterion of Suzuki's score.

The embedded liver tissues were cut to 5 *μ*M sections, stained with Ponceau after the nucleus was stained with hematoxylin, and then redyed with aniline blue liquid. Images were taken using a microscope (Olympus Corporation, Japan).

### 2.5. TUNEL Staining

For the TUNEL assay, the tissue slides were permeabilized for 10 min at 37°C and then incubated by the TdT enzyme and fluorescein-12-dUTP for 2 h at 37°C, and after stopping and washing, the TUNEL-stained tissues were visualized by a fluorescence microscopy.

### 2.6. Immunofluorescence

Liver frozen tissue sections were permeabilized by 0.2% Triton 100 for 5 min and blocked with 2% BSA solution for 30 min. After washing, the slides were incubated with primary antibodies TOM20 (Cell Signaling Technology, 1 : 100), COX2 (Abcam, 1 : 50), and COX4 (Abcam, 1 : 50) at 4°C overnight, followed by the secondary antibodies' incubation for 1 h at room temperature. For the nuclear staining, the 4',6-diamidino-2-phenylindole (DAPI) was used. The fluorescence was visualized by a confocal microscope (Olympus Corporation, Japan).

### 2.7. Measurement of Oxidative Stress-Related Markers

Liver tissue samples were homogenized in the cold phosphate buffer and centrifuged at 3000 g for 15 min, and the supernates were collected for the measurement of the oxidative stress-related markers with the relevant commercially kits and performed according to the direction of manufacture. Total protein concentration in tissue homogenate was determined by using the BCA method.

The reduced glutathione (GSH) level was assessed based on the reduction of 5, 5 dithiobis (2-nitrobenzoic acid) (DTNB) with GSH to produce a yellow compound which can be measured at 450 nm by microplate reader [26].

Glutathione peroxidase (GSH-Px) catalyzed the oxidation of GSH by hydrogen peroxide to produce GSSG. Glutathione reductase (GR) catalyzed the reduction reaction of GSSG by NADPH to reduce regenerate GSH and oxidize NADPH to NADP+. NADPH has a characteristic absorption peak at 340 nm. GSH-Px activity was calculated by measuring the rate of absorption reduction at 340 nm [27].

Hepatic lipid peroxidation (MDA) level was assessed based on the reaction of N-methyl-2-phenylindole with MDA at 45°C to produce a stable chromophore which can be measured at 586 nm [26].

Catalase (CAT) could catalyze hydrogen peroxide into water and oxygen; the residual hydrogen peroxide could oxidize the chromogenic substrate and produced the red product (N-(4-antipyryl)-3-chloro-5-sulfonate-p-benzoquinonemonoimine) which can be measured at 520 nm [28].

2′,7′-dichlorofluorescein diacetate (DCFH-DA) was used to determine the level of ROS in the liver tissue. Briefly, the liver tissue homogenate was incubated with 10 *μ*M DCFH-DA for 30 min at 37°C in dark, and the fluorescence intensity was measured by a microplate reader with wavelengths of 488 and 525 nm, respectively [29].

### 2.8. Detection of ROS by DHE on Liver Slices

The snap-frozen liver tissues were cut into 5 *μ*M slices and stained with 10 *μ*M DHE for 30 min in dark. After washing with PBS, the slices were observed with a laser scanning confocal microscopy (Olympus Corporation).

### 2.9. Mitochondrial Isolation

The mitochondria in rat liver were isolated according to the method reported before [30]. In brief, liver tissues were sliced in a cold isolation buffer A and homogenized with an automatic homogenizer. Homogenates were centrifuged at 600 g for 5 min, and the supernatants were collected for centrifugation at 11000 g for 10 min. The pallets were collected and resuspended in a cold isolation buffer B and then centrifuged at 11000 g for 10 min. Pallets were resuspended in a cold medium and centrifuged at 3500 g for 15 min. Pallets were collected, and the final mitochondria were suspended in storage buffer (4°C, pH 7.4, with 250 mM sucrose, and 10 mM HEPES-KOH) and used immediately. Buffer A contains sucrose (250 mM), EGTA (0.1%), defatted BSA (0.1%), HEPES-KOH (10 mM), 4°C, and pH 7.4. Buffer B is buffer A without BSA.

### 2.10. Mitochondrial Respiration Measurement

Mitochondrial respiration was measured as the method reported before [31]. Briefly, 1 mg isolated mitochondria were resuspended in 2 ml buffer containing sucrose (125 mM), EGTA (1 mM), defatted BSA (0.1%), MgCl2 (1 mM), KCl (100 mM), HEPES-KOH (10 mM), KH_2_PO_4_ (5 mM), rotenone (2.5 *μ*M), and pH 7.4. 5 mM succinate was used to initiate the mitochondrial respiration at 30°C, and state 3 respiration was measured after the ADP (0.5 mM) was added. State 4 respiration was measured after the ADP was exhausted. The respiratory control ratio (RCR) and ADP/O ratio in different groups were also recorded.

### 2.11. Mitochondrial Membrane Potential (MMP) Measurement

MMP was measured by rhodamine 123 (Rh123). Briefly, 1 mg isolated mitochondria were incubated with 2 ml buffer (pH 7.4) containing rotenone (2.5 *μ*M), sucrose (125 mM), KCl (65 mM), HEPES-KOH (10 mM), and Rh123 (0.2 *μ*M), at 30°C for 10 min. After these, the fluorescence in different groups was measured by a fluorescence microplate (Fluoroskan FL, Thermo). The results were showed as the fold of control group.

### 2.12. ATP Measurement

0.5 mg isolated mitochondria was homogenized with a cold ATP releasing buffer, and the supernatant was collected after centrifuged at 12000 g for 5 min. The ATP levels were measured using an ATP measurement kit (ab83355, Abcam) according to the instructions of the kit.

### 2.13. Immunoblotting Analysis

For immunoblotting analysis, the mitochondrial proteins were extracted using a mitochondrial protein extraction kit (KGP8100, Keygentec). 30 *μ*g proteins were loaded onto sodium dodecyl sulfate polyacrylamide gel electrophoresis (SDS-PAGE) and transferred onto polyvinylidene difluoride membranes (PDVF). Membranes were incubated with the primary antibodies (LC3 II, P62, Beclin1, TOM20, Parkin, PINK1, PHB2, PGAM5, and PARL (1 : 1000)) overnight at 4°C. And then, the membranes were incubated with relevantly secondary antibodies conjugated with horseradish peroxidase and visualized using the enhanced chemiluminescence detection system (Millipore). The results were showed as the fold of control.

### 2.14. Quantitative PCR

Total RNA from liver tissues was extracted using Trizol, and cDNA was synthesized using oligo (dT) prime and PrimeScript RT reagent (Takara, Japan). cDNA was amplified using SYBR Premix Ex Taq Kit (Takara, Japan) on an Applied Biosystems ABI 7500 system (CA, USA). Fold change gene expression was determined with cycle threshold (Ct) values and normalized to GAPDH expression [32]. The primer sequences in this study were showed in Table 1.

### 2.15. mtDNA Copy Number

Total DNA in liver tissues were extracted by phenol-chloroform method. Real-time PCR using ABI 7500 System was applied to quantify mitochondria DNA (mtDNA) copy number. The primers for mtDNA were forward (CGAAAGGACAAGAGAAATAAGG) and reverse (CTGTAAAGTTTTAAGTTTTATGCG). *β*-globulin was used as genomic DNA control, and the primers for *β*-glubin were forward (CAACTTCATCCACGTTCACC) and reverse (GAAGAGCCAAGGACAGGTAC).

### 2.16. Transmission Electron Microscopy (TEM)

For TEM assay, the liver tissues (1 mm^3^) were fixed with 2.5% glutaraldehyde and then postfixed with 1% osmium tetroxide for 1 h, dehydrated in graded acetone, and embedded in Epon-Araldite resin. Next, thins (80 nm) were cut by ultramicrotome instrument (Leica, Germany). Then, slices were poststained with 2% aqueous uranyl acetate and Reynold's lead citrate. Preparations were imaged at the HT7700 Electron Microscopy (Hitachi, Japan).

### 2.17. Statistical Analysis

Results were analyzed by a GraphPad Prism 5.0 software. Data were showed as mean ± SD from at least three experiments. Student's *t*-test (two-tailed) was used for the comparisons between two groups. ANOVA followed by the post hoc Bonferroni test was used for multiple comparisons. *P* values below 0.05 were considered statistically significant.

## 3. Results

### 3.1. FN Protected Liver from I/R-Induced Injuries

**Figure 1:**
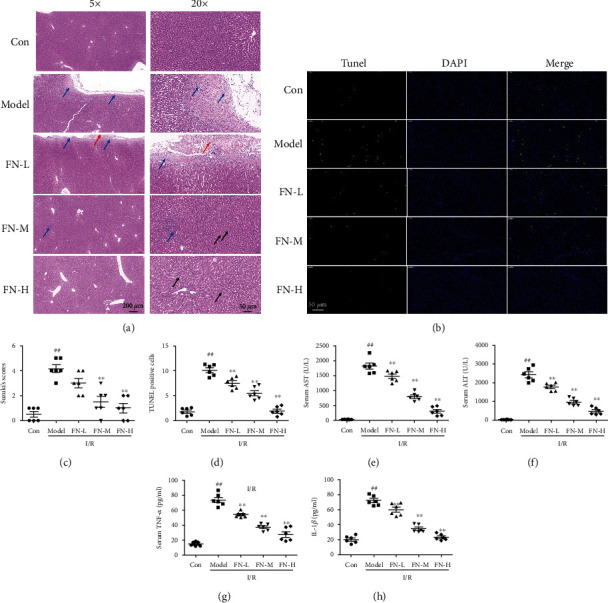
The protective effects of FN were evaluated. (a) HE staining. Left: 5×; right: 20×. Red arrow showed the acidophilia, black arrows showed the circular vacuoles, and blue arrow showed the lymphocytic infiltration. (b) Representative TUNEL staining images (20×). (c). Suzuki scores were calculated from HE staining. (d) TUNEL positive staining cells were calculated from TUNEL staining. (e) AST levels in the serum after different treatments. (f) ALT levels in the serum after different treatments. (g) TNF-*α* levels in the serum after different treatments. (h) IL-1*β* levels in the serum after different treatments. ^##^*P* < 0.01 vs. control group; ^∗∗^*P* < 0.01 vs. model group. *N* = 6 in every group.

The liver histopathological changes in Figure 1(a) showed that I/R-induced extensive necrosis which characterised by the sinusoidal congestion and cell swelling. And large area of capsule thickening and amounts of lymphocyte infiltration were observed in the model group. The damage degree was calculated using Suzuki criterion scores, which showed that model group had a higher score compared with the control group (*P* < 0.01, Figure 1(c)). FN treatments significantly improved the liver histopathological injuries compared with the model group (*P* < 0.01). TUNEL staining showed the I/R-induced cell apoptosis in the liver which appeared by the TUNEL positive staining in the model group (Figures 1(b) and 1(d)). In the FN treatment groups, the TUNEL positive staining was decreased significantly in a dose dependent manner (*P* < 0.01). Serum levels of AST (Figure 1(e)), ALT (Figure 1(f)), TNF-*α* (Figure 1(g)), and IL-1*β* (Figure 1(h)) were measured. As the results showed, the levels of AST, ALT, TNF-*α* and IL-1*β* in serum were both increased significantly in the rats subjected to I/R operation (*P* < 0.01, compared with control group). After FN treatments, the levels of AST, ALT, TNF-*α*, and IL-1*β* were decreased and showed significantly difference with that in model group (*P* < 0.01). These results suggested that FN had protective effects against I/R-induced liver injuries.

### 3.2. FN Protected Liver from I/R-Induced Mitochondrial Dysfunction

**Figure 2:**
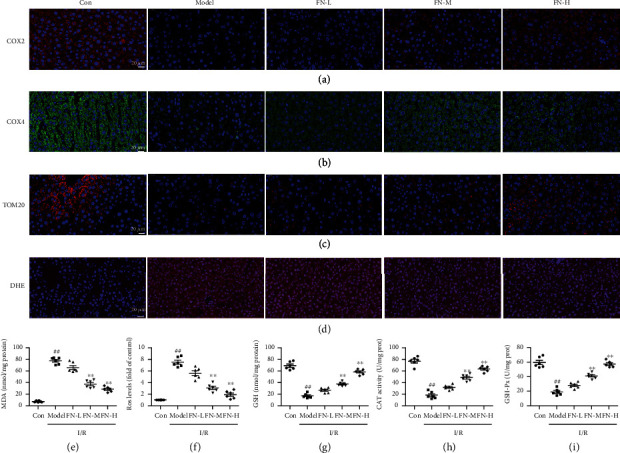
FN inhibited the I/R-induced mitochondrial dysfunction. Liver frozen tissue sections (5 *μ*M) were prepared by a freezing microtome and then stained by respective antibodies or staining. (a) Representative immunofluorescence results for COX2 which were detected by specific COX2 primary antibody and corresponding secondary antibody (60×). (b) Representative immunofluorescence results for COX4 which were detected by specific COX4 primary antibody and corresponding secondary antibody (60×). (c) Representative immunofluorescence results for TOM20 which were detected by specific TOM20 primary antibody and corresponding secondary antibody (60×). (d) Representative immunofluorescence results for DHE staining (10 *μ*M) (40×). (e) ROS level in the liver tissues was measured by a DCFH-DA kit as the protocol direction. The results were showed as the fold of control. The liver tissue homogenate was prepared for the measurements of (f) MDA, (g) GSH, (h) CAT, and (i) GSH-Px as the protocol direction. Protein concentrations in every group were measured by a BCA protein assay kit.^##^*P* < 0.01 vs. control group; ^∗∗^*P* < 0.01 vs. model group. *N* = 6 in every group.

To evaluate the effects of FN on I/R-induced mitochondrial dysfunction, the mitochondria-related makers were measured. As the results showed in Figures 2(a) and 2(b), I/R induced the decrease expression of COX2 and COX4, which were main components of the mitochondrial respiratory chain. We also observed that the mitochondrial number was decreased after I/R treatment which characterised by the TOM20 staining (Figure 2(c)). FN treatments significantly restored the COX2 and COX4 positive staining and increased the number of mitochondria.

ROS levels were also measured to evaluate the oxidative burden induced by I/R. Figure 2(d) was the results of DHE staining which is a fluorescent probe for the detection of ROS generation and specific for superoxide. As the results showed, I/R induced increase of red fluorescence intensity which indicated the production of superoxide. In FN treatment (FN-M and FN-H) groups, the intensity of red fluorescence was both decreased which indicated FN inhibited the superoxide production induced by I/R. And the ROS levels were also quantitative measured by DCFH-DA kit. Compared with the ROS levels in sham group, the ROS level was increased about 7.5-fold of control in model group. In the FN treatment groups, the ROS levels were decreased about 3-fold and 1.9-fold of control in FN-M and FM-H group, respectively (Figure 2(e)).

As the results showed in Figure 2(f), compared with the sham group, the level of MDA in model group was 77.32 ± 5.66 nmol/mg prot, which significantly higher than that in the sham group (7.33 ± 1.35 nmol/mg prot), indicating the lipid peroxidated by ROS. After FN treatment, especially the higher dosages, the MDA levels were significantly lowered (28.35 ± 4.39 nmol/mg prot in FN-H vs. 77.32 ± 5.66 in model group, *P* < 0.01).

The antioxidant proteins (GSH, CAT, and GSH-Px) were also measured. As the results showed in Figure 2(g), GSH level was significantly decreased in the model group (17.32 ± 3.92 nmol/mg prot vs. 68.98 ± 6.7 nmol/mg prot, *P* < 0.01), and FN treatments significantly restored the GSH levels (58.38 ± 4.54 nmol/mg prot in FN-H vs. 17.32 ± 3.92 nmol/mg prot in model group, *P* < 0.01). The activity of CAT was also inhibited in the model group (18.3 ± 5.96 U/mg prot vs. 76.88 ± 7.5 U/mg prot, *P* < 0.01) and rescued by the FN treatment (63.25 ± 4.65 U/mg prot in FN-H vs. 18.3 ± 5.96 U/mg prot in model group, *P* < 0.01). The same changes were also observed in the activities of GSH-Px, which showed in Figure 2(i). These results suggested that FN restored the mitochondrial dysfunction and inhibited the oxidative stress induced by I/R in the liver.

### 3.3. FN Inhibited the Energy Metabolism Failure Induced by I/R

**Figure 3:**
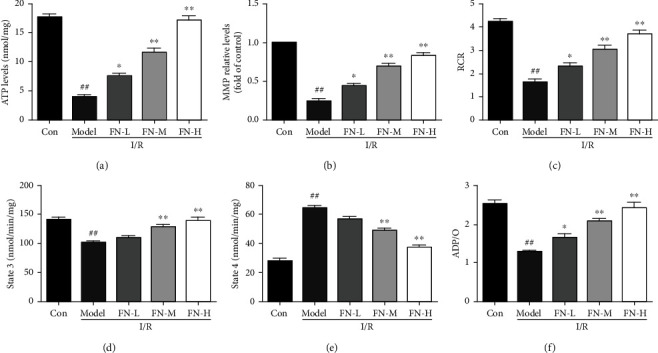
Effects of FN on the mitochondrial energy metabolism. Rat liver mitochondria were isolated after different treatments in different groups as the method showed in the materials and methods section. (a) Effects of FN on the ATP levels after different treatments. (b) Effects of FN on MMP levels, and the results showed as the fold of that in control group. (c) RCR results. (d) State 3 levels. (e) State 4 levels. (f) ADP/O levels. ^##^*P* < 0.01 vs. control group; ^∗∗^*P* < 0.01 vs. model group. *N* = 6 in every group.

The mitochondrial dysfunction will induce energy metabolism failure; thus, the energy metabolism-related indicators were measured in the isolated liver mitochondria. As the results showed in Figure 3, I/R induced ATP synthesis impairment (Figure 3(a)), MMP consumption (Figure 3(b)), and respiration dysfunction which indicated by RCR (Figure 3(c)), state 3 (Figure 3(d)), state 4 (Figure 3(e)), and ADP/O (Figure 3(f)). In the FN treatment groups, the drop of MMP and ATP levels was increased, and mitochondrial respiration was improved in both dosage treatment groups. These results suggested that FN improved the energy metabolism failure induced by I/R.

### 3.4. FN Induced Parkin/PINK1-Dependent Mitophagy in Liver

**Figure 4:**
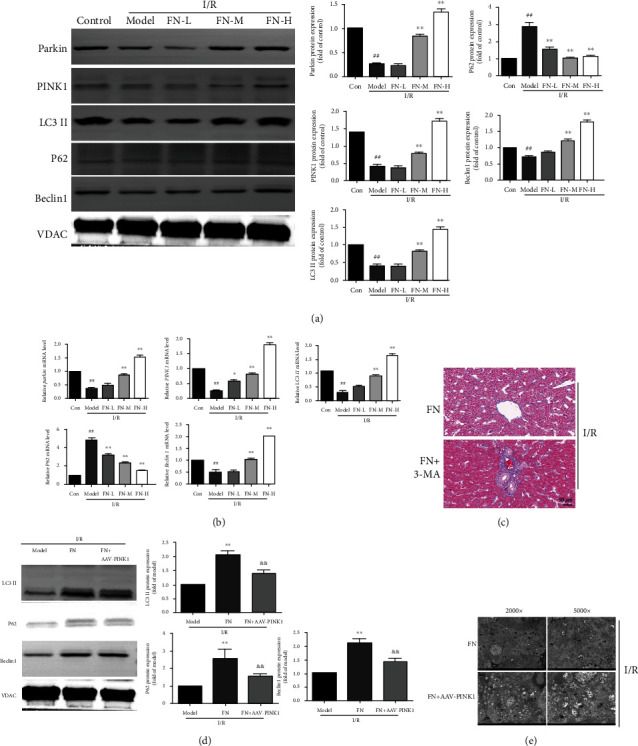
Effects of FN on the Parkin/PINK1 regulating mitophagy. (a) The protein expression levels of Parkin, PINK1, LC3 II, p62, and Beclin1 were measured by western blotting. (b) The mRNA expression levels of Parkin, PINK1, LC3 II, p62, and Beclin1 were measured by RT-PCR. (c) 3-Methyladenine (3-MA), a widely used inhibitor of autophagy, was used together with FN, and Masson staining was used to evaluate the liver injury (60×). (d) The protein expression levels of LC3II, P62, and Beclin1 were measured after AAV-PINK1 treatment. (e) Representative pictures of TME; autolysosome was marked as ASS. ^##^*P* < 0.01 vs. control group, ^∗∗^*P* < 0.01 vs. model group, and ^&&^*P* < 0.01 vs. FN group. *N* = 6 in every group.

Mitophagy plays an important role in regulating mitochondrial function; thus, we observed the mitophagy-related proteins and genes. As the results showed in Figure 4(a), the protein expression of Parkin and PINK1 was both decreased in the model group, as well as the levels of mitophagy's markers (LC3 II and Beclin1), and the protein expression of P62 was increased, suggesting that I/R inhibited the Parkin/PINK1-mediated mitophagy pathway. In FN treatment groups (FN-M and FN-H), the protein expression of Parkin, PINK1, LC3 II, and Beclin1 was increased significantly compared with that in the model group. The mRNA expression of Parkin, PINK1, LC3 II, P62, and Beclin1 results also showed that FN treatments regulated the mitophagy-related gene expression (Figure 4(b)). These results suggested that FN regulated the mitophagy in the hepatic I/R model.

Next, we used 3-methyladenine (3-MA), a widely used inhibitor of autophagy, to evaluate the protective effects of FN whether though regulating mitophagy. As expected, 3-MA abolished the effects of FN in inhibiting liver fibrosis which measured by Masson staining (Figure 4(c)). To further explore the role of Parkin/PINK1 in the mitophagy regulating effects of FN, an adeno-associated virus was transformed to hepatocyte-specifically knockdown PINK1 in liver. As the results showed in Figure 4(d), AAV-PINK1 significantly inhibited the regulating effects of FN on the mitophagy-related markers (LC3 II, P62, and Beclin1). To explore the effect of PINK1 downexpression on autophagy in hepatic I/R, electron microscopy was used (Figure 4(e)). Notably, AAV-PINK1 significantly diminished the number of autolysosome in hepatic I/R tissues (10 in FN group vs. 3 in FN + AAV-PINK1 group). These results suggested that FN induced mitophagy through the Parkin/PINK1 pathway.

### 3.5. The Mitochondrial Protective Effect of FN Was Dependent on PINK1

**Figure 5:**
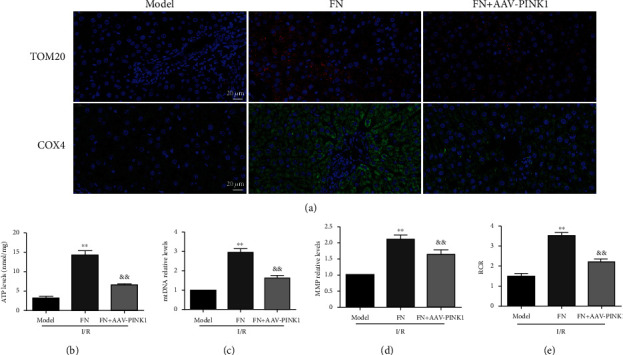
PINK1 played an important role in regulating FN's protective effects. (a) Representative immunofluorescence results for COX4 and TOM20 (60×). (b) ATP levels. (c) mtDNA relative levels. (d) MMP relative levels; the results showed as the fold of control. (e) RCR. ^∗∗^*P* < 0.01 vs. model group; ^&&^*P* < 0.01 vs. FN group. *N* = 6 in every group.

Further, we evaluated the protective effects of FN whether through PINK1. As the results showed in Figure 5(a), the elevated role of FN on the expression of TOM20 and COX4 was abolished by PINK1 knockdown (FN + AAV-PINK1 vs. FN). As compared with the FN treatment group, the improvement effects of FN on ATP synthesis impairment (Figure 5(b)), mtDNA expression level (Figure 5(c)), MMP consumption (Figure 5(d)), and respiration dysfunction (Figure 5(e)) were also inhibited by PINK1 knockdown. These results suggested that the mitochondria regulating effect of FN was dependent on PINK1.

### 3.6. FN Regulated Parkin/PINK1-Dependent Mitophagy through PHB2

**Figure 6:**
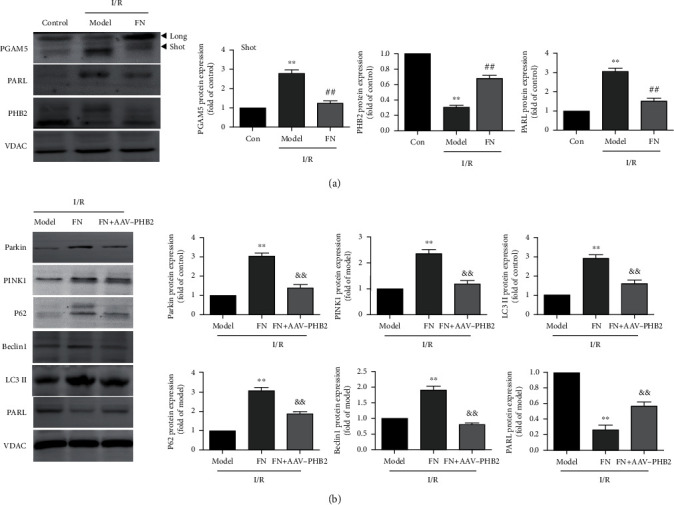
FN regulated the PHB2-related protein expression. (a) The protein expression levels of PGAM5 (long and shot), PARL, and PHB2 were measured with their special antibodies. The calculation results were showed in the right as bar graph. (b) PHB2 knockdown abolished the regulation effects of FN on the protein expression of Parkin, PINK1, P62, Beclin1, LC3 II, and PARL. ^##^*P* < 0.01 vs. control group, ^∗∗^*P* < 0.01 vs. model group, and ^&&^*P* < 0.01 vs. FN group. *N* = 6 in every group.

Next, we explored which protein was targeted by FN in regulating Parkin/PINK1-dependent mitophagy. According to the stabilization and aggregation effects of PHB2 on Parkin/PINK1 in mitochondria, PHB2 and its downstream were measured. As the results showed in Figure 6(a), FN increased the protein expression level of PHB2 which was decreased by I/R treatment (*P* < 0.01). PARL levels and the shot PGAM5 protein levels were increased after I/R, which compared with the control group. In the FN treatment group, the PARL and shot PGAM5 protein expression levels were decreased compared with the model group (*P* < 0.01). To further clear and definite the role of PHB2 in FN regulating mitophagy, an adeno-associated virus transformed to hepatocyte-specifically knockdown PHB2 in liver was used. As the results showed in Figure 6(b), compared with FN group, the increase effects of FN on Parkin and PINK1 protein expression were inhibited by PHB2 knockdown (*P* < 0.01), suggesting FN regulated Parkin/PINK1 through PHB2. Also, PHB2 knockdown abolished the regulating effects of FN on P62, Beclin1, and LC3II protein expression (*P* < 0.01), suggesting that FN regulated mitophagy through PHB2. The decreased effect of FN on PARL expression was also abolished by PHB2 knockdown (*P* < 0.01). These results suggested that FN regulated Parkin/PINK1-dependent mitophagy through PHB2.

### 3.7. The Mitochondrial Protective Effect of FN Was Dependent on PHB2

**Figure 7:**
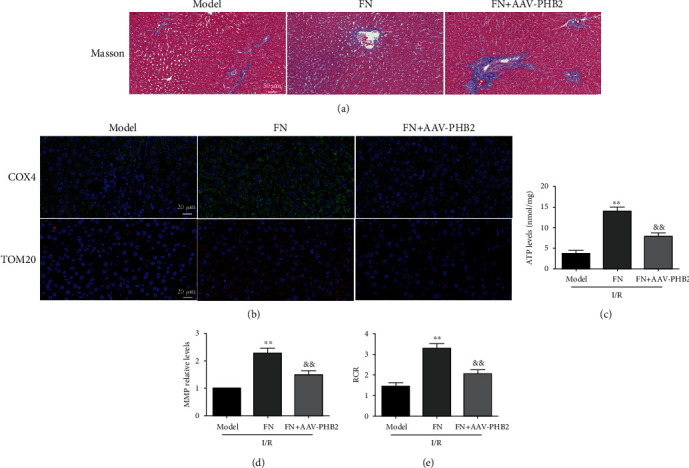
The role of PHB2 in FN performing liver protective effects. (a) Masson staining (20×). (b) Representative immunofluorescence results for COX4 and TOM20 (60×). (c) ATP levels. (d) MMP relative levels; the results showed as the fold of control. (e) RCR. ^∗∗^*P* < 0.01 vs. model group; ^&&^*P* < 0.01 vs. FN group. *N* = 6 in every group.

The mitochondria-related indicators were measured to evaluate the effect of PHB2 in FN's protective effect. As shown in Figure 7(a), PHB2 knockdown significantly increased the liver fibrosis level in the FN + AAV-PHB2 group, which compared with the FN group (Figure 7(a)). PHB2 knockdown also inhibited the protein expression levels of COX4 and TOM20 in liver tissues which were both increased by FN treatment (Figure 7(b)). The improvement effects of FN on ATP synthesis impairment (Figure 7(b)), MMP consumption (Figure 7(c)), and respiration dysfunction which indicated by RCR (Figure 7(d)) were also inhibited by PHB2 knockdown. These results suggested that FN performed its protective effects against I/R through PHB2 pathway.

## 4. Discussion

In previous studies, the hepatocyte protection effects of FN had been partly investigated. FN inhibited FFA-stimulated lipid accumulation through denosine monophosphate-activated protein kinase (AMPK)/transcription factor EB- (TFEB-) mediated lysosome biogenesis [33]. In a carbon tetrachloride-induced liver injury model, FN performed its hepatocyte protection effects through reducing TNF, NF-*κ*B, and Toll-mediated inflammatory [19]. In a ritonavir-induced liver injury model, FN showed its protective effects through inhibiting oxidative stress, inflammation, and apoptosis [34]. Additional, FN also had protective effects against epithelial cell injury, endothelial dysfunction, and brain injuries [21, 35, 36]. These studies enlightened that FN has multiple biological effects and deserved to further worth in-depth study. In consideration of the hepatocyte protection of FN in other models, we hypothesized that FN had protective effects against I/R-induced liver injury. In this study, FN protected liver from I/R-induced histopathological injury, improved the liver function which indicated by decreasing the AST and ALT levels, inhibited the apoptosis, and reduced the inflammatory. These results confirmed the protective effects of FN against I/R-induced liver injury. Further, the possible mechanism was investigated to explain these effects.

Hepatic ischemia induces anaerobic glycolysis and acidic metabolites accumulation, thus disequilibrating the mitochondrial homeostasis, resulting in mitochondrial damage [37]. ROS is the main byproduct in energy metabolism, cleared by the antioxidant proteins in mitochondria, while ROS is overproduced, and antioxidants are destroyed during reperfusion, thus aggravate the mitochondrial damages [38]. In our study, we observed that the ROS and MDA levels were increased in the I/R group, together with the decrease of GSH, CAT, and GSH-Px activities. The results also showed that I/R induced mitochondrial metabolic dysfunction indicated by the reduction level of ATP and respiration dysfunction, suggesting that I/R induced mitochondrial dysfunction in the liver. FN treatment improved the mitochondrial function by reversing these changes.

Mitophagy, a type of autophagy in mitochondria, can clear the dysfunctional mitochondria and prevent the accumulation of mitochondrial byproducts. Following prolonged ischemia and subsequent reperfusion, the extent of mitochondrial injury inhibits the scavenging activity of autophagy target I/R-damaged mitochondria [10]. Impaired or insufficient mitophagy induced uncontrolled ROS production, mitochondrial DNA mutation, metabolic dysfunction, and ultimately cell death [39]. According to those, rescuing the mitophagy activity after reperfusion or during the ischemia period can protect I/R-induced liver injury. In this study, we found that I/R-induced mitophagy-related protein (LC3 II, P62, and Beclin1) downexpressions were rescued by FN treatment, suggesting that FN protected liver I/R injury might through increasing mitophagy in mitochondria.

PINK1/Parkin-mediated mitophagy is one of the most important autophagy pathways and is involved in eliminating the dysfunctional mitochondria during hepatic I/R-induced injury [40]. Parkin, a cytosolic RBR (RING-between-RING) E3 ubiquitin ligase, contains a Ub1 (N-terminal ubiquitin-like) domain. Upon steady state conditions, Parkin adopts an autoinhibited conformation, and the cytosolic ligase is inactivated [41]. Upon mitochondrial depolarization, PINK1 accumulates on the OMM of dysfunctional mitochondria and recruits Parkin from the cytosol onto OMM and activates its E3 ligase activity, thus Parkin ubiquitinates various outer-membrane proteins and induces the initiation of mitophagy [42]. However, little is known regarding the role of PINK1/Parkin-mediated mitophagy in FN-induced hepatocyte protection. In this study, we found that PINK1 and Parkin expression levels in mitochondria were inhibited in the hepatic I/R rat model, and FN increased the expression of PINK1 and Parkin in a dose dependent manner. Further, we also found PINK1 knockdown significantly inhibited the effects of FN in regulating mitophagy as well as the mitochondria protective effects. These results suggested that FN induced mitophagy though regulating PINK1/Parkin-dependent pathway.

Next, we tried to explore how FN regulated PINK1/Parkin-dependent mitophagy pathway. In mammalian cells, prohibitin 2 (PHB2) plays as an important receptor for PINK1-mediated mitophagy [43]. PARL, a glimpse at intramembrane proteolysis in the mitochondrial inner membrane, had been reported to negatively regulates PINK1/Parkin-mediated mitophagy. PARL can cleave several substrates including PGAM5, which stabilify the PINK1 with a full-length PINK1 style, thus induces the initiation of mitophagy [44, 45]. When PGAM5 is cleaved by PARL to generate the short form of PGAM5, the PGAM5-mediated PINK1 insertion into mitochondria was impaired and leads to the degradation of PINK1, thus inhibits the mitophagy [46]. PHB2 can bind with PARL and PGAM5 in the long form to stabilize PINK1 in damaged mitochondria in OMM, thus induces the mitophagy [47]. According to the effects of FN in regulating PINK1/Parkin-mediated mitophagy, we further investigated whether PHB2/PARL/PGAM5 was involved in this process. As our results showed, I/R decreased the expression of PHB2 and increased the expression of PARL, together with the increased expression of PGAM5 in the shot form, suggesting that the reduction of PHB2 induced by hepatic I/R might contribute to the mitophagy dysfunction. As expected, FN treatment significantly rescued the PHB2 expression and decreased the PARL and shot-PGAM5 expression, suggesting PHB2 might participate in the regulating effect of FN on PINK1/Parkin-regulated mitophagy. To confirm the crosstalk between PHB2 and PINK1/Parkin in regulating mitophagy by FN, adeno-associated virus was used to knockdown PHB2. Interestingly, PHB2 knockdown significantly reduced the effects of FN on the PINK1/Parkin-related mitophagy and also abolished the protective effects against I/R-induced mitochondria dysfunction. These results suggested that FN performed its effects on PINK1/Parkin-related mitophagy through PHB2.

## 5. Conclusion

In summary, we confirmed an isoflavone compound, FN, could protect I/R-induced liver injury though regulating PINK1/Parkin-mediated mitophagy pathway. Further, we also found that PHB2-PARL-PGAM5 played an important role in FN-mediated PINK1 accumulation in mitochondria. These results suggested that PHB2-PINK1-related mitophagy might be a possible target for I/R-induced liver injury. The compounds targeting this pathway may be beneficial for the treatment of ischemia-related liver diseases. Furthermore, it also exposed that FN might be a mitochondrial protective agent or food/nutrition supplement for the clinical treatment of hepatic I/R injury. Our findings provided novel insights into the potential prevention strategies of FN and its underlying mechanisms.

## Figures and Tables

**Table 1:** Primer sequences of mitophagy-related genes.

*Genes*	Forward (5′-3′)	Reverse (5′-3′)
*Parkin*	GACTGCTTCCACTTGTACTGT	AGCTCTTTAATCAGGGAGTTGG
*Pink1*	TCTTAAGCAAAATGAGCCAGGA	CTACCGCCTGAACTGTTGAAG
*LC3 II*	TCGAACAAAGAGTGGAAGATGT	TCTCACCCTTGTATCGCTCTAT
*Beclin1*	CAATGTCTTCAATGCGACCTTC	GGCAGCATTGATTTCATTCCAT
*P62*	CGAAATGCAGAGAAAAGACCAC	ATTCCATGAGTTTCCGGTTGAT
*GAPDH*	GGAGTCTACTGGCGTCTTCAC	ATGAGCCCTTCCACGATGC

## Data Availability

The data used to support the findings of this study are available from the corresponding authors upon request.
